# Reference Values for Macronutrients in Human Milk: the Mothers, Infants and Lactation Quality (MILQ) Study

**DOI:** 10.1016/j.advnut.2025.100501

**Published:** 2025-10-28

**Authors:** Jack I Lewis, Daphna K Dror, Daniela Hampel, Gilberto Kac, Christian Mølgaard, Sophie E Moore, Janet M Peerson, Sophie Hilario Christensen, M Munirul Islam, Daniela de Barros Mucci, Amanda C Figueiredo, Adriana Divina de Souza Campos, Mehedi Hasan, Lindsay H Allen, The MILQ Study Consortium, Lindsay H Allen, Sophie E Moore, Gilberto Kac, Kim F Michaelsen, Christian Mølgaard, M Munirul Islam, Maria Andersson, Setareh Shahab-Ferdows, Sophie H Christensen, Jack I Lewis, Janet M Peerson, Xiuping Tan, Daphna K Dror, Andrew M Doel, Daniela de Barros Mucci, Bruna C Schneider, Farhana Khanam, Adriana Divina de Souza Campos, Gabriela Torres Silva, Fanta Nije, Mehedi Hassan, Amanda C Figueiredo, Daniela Hampel

**Affiliations:** 1 Department of Nutrition, Exercise and Sports, Faculty of Science, University of Copenhagen, Copenhagen, Denmark; 2 United States Department of Agriculture, Agricultural Research Service, Western Human Nutrition Research Center, Davis, CA, United States; 3 Institute for Global Nutrition, Department of Nutrition, University of California, Davis, CA, United States; 4 Nutritional Epidemiology Observatory, Josué de Castro Nutrition Institute, Federal University of Rio de Janeiro, Rio de Janeiro, Brazil; 5 Medical Research Council Unit, The Gambia at London School of Hygiene & Tropical Medicine, Fajara, The Gambia; 6 Department of Women and Children's Health, King's College London, London, United Kingdom; 7 Center for Clinical Research and Prevention, Copenhagen University Hospital—Bispebjerg and Frederiksberg Hospital, Copenhagen, Denmark; 8 Nutrition and Clinical Services Division, International Centre for Diarrhoeal Disease Research, Bangladesh, Dhaka, Bangladesh; 9 Department of Basic and Experimental Nutrition, Rio de Janeiro State University, Rio de Janeiro, Brazil

**Keywords:** Human milk, lactation, macronutrients, protein, fat, carbohydrate, reference values, milk nutrient concentration, infant nutrient requirements

## Abstract

This second article in the series establishing reference values (RVs) for nutrients in human milk describes RVs for protein, carbohydrate, fat, and energy. To establish RVs, the mothers, infants, and lactation quality (MILQ) and early-MILQ studies collected human milk samples throughout the first 8.5 mo of lactation in 1242 well-nourished women in Bangladesh, Brazil, Denmark, and The Gambia. Macronutrients were measured by near-infrared spectroscopy. Protein concentrations decreased from 12.4 g/L at 4–17 d to a 7.7–7.9 g/L plateau by 4–5 mo. Carbohydrate concentrations were stable throughout lactation, ranging from 68.2 to 70.1 g/L. Fat concentrations decreased from 37.0 g/L at 4–17 d to 31.2–32.8 g/L after 2–3 mo. Energy density mirrored fat trends, decreasing from 665 kcal/L at 4–17 d to 597–602 kcal/L by 3–4 mo. Compared with estimates used by the Institute of Medicine (IOM)--renamed the National Academy of Medicine (NAM) in 2015--to set nutrient intake recommendations for infants, MILQ values were ∼90% of concentrations used for carbohydrate and energy, and 70%–80% for protein and fat. Total daily median intakes (concentrations × milk volumes) from 1 to 6 mo were on par with IOM adequate intakes (AIs) for carbohydrate and energy, 65% of the AI for protein, and 84% of the AI for fat. These RVs offer a critical resource for understanding the nutritional contributions of human milk and informing public health practices to support infant growth and development.

**Statement of significance**

This article in the series on reference values for nutrients in human milk in the mothers, infants and lactation quality study describes the concentrations of macronutrients and energy across the first 8.5 mo of lactation. Because milk volume was measured simultaneously, infants' total intake of each macronutrient was also measured. Protein and fat concentrations and intakes were lower than those used to set the Institute of Medicine’s (IOM) recommended intakes for infants and lactating women, suggesting that these values may need updating.

## Introduction

Nutrient intakes in the first months of life maintain physiological processes, including growth, and influence infants’ lifelong health trajectory [1]. Despite the critical role of human milk, research on its composition has been complicated by sample collection methods, analytical techniques, and interindividual differences, which can be attributed to maternal diet, mammary gland physiology, maternal health, and environmental factors [2]. An improved understanding of the sources of variability and refined analytical techniques allows for more accurate quantification of nutrients in human milk.

The macronutrients in human milk comprise proteins, carbohydrates, and fats. Exclusively breastfed infants derive most of their energy from fats and carbohydrates (40%–50% each), with proteins contributing <10% of total energy in mature milk [1,3]. In addition to promoting growth, amino acids and fatty acids contribute to metabolic processes, immunity, and infant development [4]. Although macronutrients in human milk have been a subject of study for decades, relatively few studies have measured milk composition across stages of lactation, and fewer still have measured milk volume at the same time.

The mothers, infants, and lactation quality (MILQ) and early MILQ (E-MILQ) studies were designed to develop reference values (RVs) for nutrient concentrations in the milk of healthy mothers. The study design and methods have been described in detail previously [5,6]. In brief, human milk samples and anthropometric, dietary, biochemical, and other data were collected from mother–infant dyads at 4 study sites (Bangladesh, Brazil, Denmark, and The Gambia). Sites were chosen based on the following criteria: population of well-nourished women of childbearing age; cultural practice of exclusive and prolonged breastfeeding based on national data; minimal maternal nutrient supplementation or dietary fortification; investigators experienced in perinatal research; and geographical diversity.

Data collection occurred from 0 to 1 mo [E-MILQ, with sample collection at 4–17 d (E1) and 18–31 d (E2)], then 3 times between 1 and 8.5 mo lactation [1–3.49 mo (M1), 3.5–5.99 mo (M2), and 6–8.5 mo (M3)]. Study visits in E-MILQ and MILQ were divided in equal increments to ensure continuous data collection representative of the full study duration. The 2 study visits in E-MILQ enabled 14-d collection of saliva samples for measuring milk volume using the deuterium dilution method. An additional feature of the MILQ visit timing was to distinguish periods of exclusive breastfeeding (required through M1 and encouraged through M2) and mixed feeding. Macronutrients were analyzed by near-infrared spectroscopy on a SpectraStar XT Neonatal Analyzer (Unity Scientific), which also calculated energy density. Near-infrared spectroscopy is the current analytical method of choice due to its accuracy and convenience in measuring milk macronutrients simultaneously, and is also used to measure milk composition in hospital settings [7]. For convenience, if 1 macronutrient had an outlying value, all were coded as missing. A total of 3057 samples were analyzed (2483 MILQ, 574 E-MILQ), of which 15 were removed for implausible values (14 MILQ, 1 E-MILQ), and 306 for not meeting inclusion criteria (254 MILQ, 52 E-MILQ), for a total included sample size of 2736 (2215 MILQ, 521 E-MILQ).

This article presents the RVs for protein, carbohydrate, fat, and energy. It is the second in a series of 7 in this supplement, which includes the introduction, RVs for water-soluble vitamins, fat-soluble vitamins, and minerals, as well as milk volumes at each time point. The supplement concludes with an article on the applications of the research. Results are compared with IOM values for nutrient concentrations in milk and with adequate intake (AI) values (TABLE 1, TABLE 2). Of note, the IOM was renamed the National Academy of Medicine (NAM) in 2015. Since nutrient recommendations for protein, carbohydrate, and fat were published prior to this change we will refer to the organization as the IOM for these nutrients, Revised energy recommendations were published by the NAM in 2023. The graphs provided in this article show the concentration of each nutrient in milk by site and day of lactation, and percentiles by day of lactation. Supplementary tables include estimated percentile values of nutrient concentration by month postpartum (Supplemental Table 1) as well as median total nutrient intake by study visit (1–3.49 mo, 3.5–5.99 mo, 6–8.5 mo) (Supplemental Table 2). The introductory article in this series provides a description of how the graphs and values were constructed [6].

**TABLE 1:** Comparison of MILQ (1–6 mo) and IOM values (0–6 mo) for macronutrient concentrations in human milk.

Nutrient	MILQ^1^ median	MILQ IQR	IOM	MILQ as % IOM
Protein (g/L)	8.5	(7.0–10.3)	11.7	73
Carbohydrate (g/L)	69	(67–71)	74	93
Fat (g/L)	32	(24–44)	40	80
Energy (kcal/L)	607	(520–704)	650	93

Abbreviations: IOM, Institute of Medicine; MILQ, mothers, infants, and lactation quality study; NAM, National Academy of Medicine.

1 MILQ values are pooled median concentrations from 1 to 6 mo.

**TABLE 2:** Comparison of MILQ pooled median macronutrient intake in exclusively breastfed infants from 1 to 6 mo with IOM adequate intakes (AIs)^1^.

Nutrient	MILQ	IOM AI	%AI
Protein (g/d)	7.1	9.1	65
Carbohydrate (g/d)	57.1	60	95
Fat (g/d)	26.0	31	84
Energy (kcal/d)	487.5	438–645^2^	76%–111%

Abbreviations: IOM, Institute of Medicine; MILQ, mothers, infants, and lactation quality study; NAM, National Academy of Medicine.

1 AIs for 0–6 mo.

2 Not considered an AI, depends on infant sex and weight.

## Protein

### Background

Proteins in human milk consist of ∼ 60% whey and 40% casein, as well as mucins present in the membranes of the milk fat globules [8]. Although protein is an energy-yielding nutrient that provides amino acids to support growth and development, it is also a bioactive compound with functions such as antimicrobial activity, nutrient absorption, and intestinal development [4,8,9]. Whey proteins are highly digestible and provide amino acids for growth, whereas casein proteins slow gastric emptying to enhance nutrient absorption [10]. The majority of amino acids in human milk are protein bound; however, 2%–5% are present as free amino acids [10,11]. Beyond nutrition, free amino acids in human milk serve functional roles in gut development, neurodevelopment, and immune support [10].

Although the prevalence of protein-energy malnutrition among women of childbearing age remains a global concern [12], milk protein concentration is physiologically regulated and well conserved except in cases of severe malnutrition [13]. There is some evidence that human milk total protein concentration is correlated with maternal protein intake [14,15], but this has not been demonstrated consistently [16,17].

### Results

**FIGURE 1:**
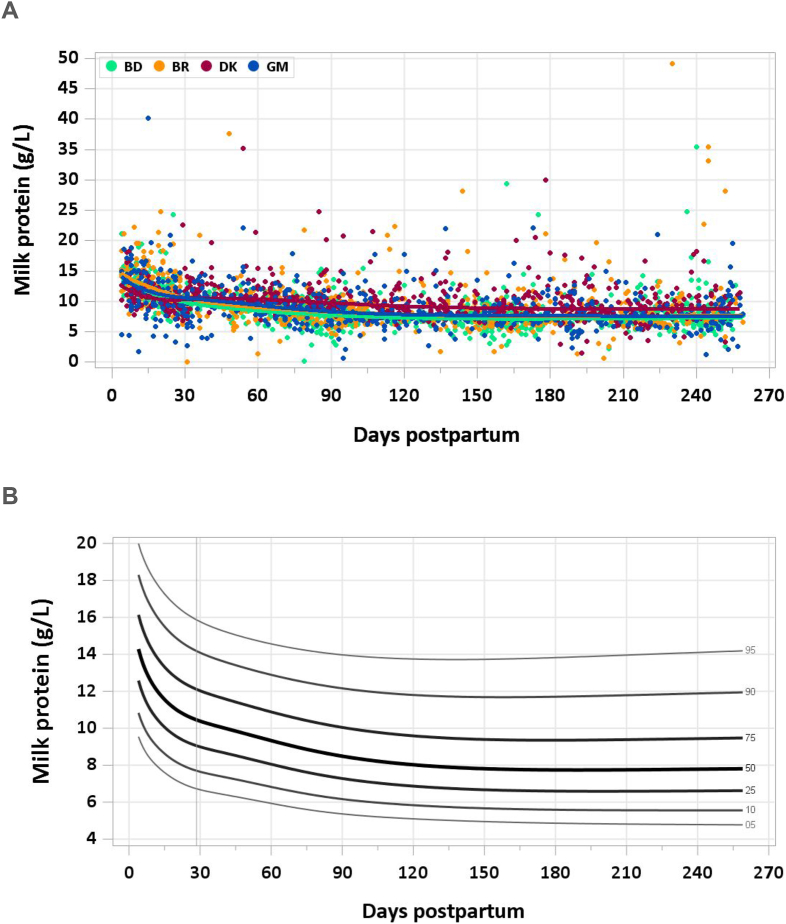
(A) Distribution of human milk protein concentrations. BD, Bangladesh; BR, Brazil; DK, Denmark; GM, The Gambia. (B) Percentile curves for protein concentration in human milk.

The protein concentration decreased steadily from a median of 12.4 g/L at 4–17 d to 9.8 g/L at 1–2 mo and 8.2 g/L at 3–4 mo. A plateau of 7.7–7.9 g/L was reached by 4–5 mo and continued throughout the study period (Figure 1A and B). The pooled median total protein intake from 1 to 6 mo was 7.13 g/d.

### Comparison with published values

The Institute of Medicine (IOM) recommends an AI of 9.1 g/d for infants from 0 to 6 mo, or 1.52 g/kg/d based on the reference weight of 6 kg for an infant aged 2–6 mo [18]. This is calculated based on the assumed average human milk intake of 0.78 L/d and an average protein concentration of 11.7 g/L in several studies in which sample size was ≥10 [[19], [20], [21], [22]]. It is worth noting that the IOM recommendation includes the period from 0 to 1 mo, when milk protein concentration is higher. Data from the present study suggest that an intake of 7.1 g/d may be adequate, particularly for infants aged >1 mo. However, the median protein concentrations measured in the MILQ study are in a similar range to those reported in the literature: 8–10 g/L [23], 7.3–16.0 g/L [24], or 7–10 g/L when restricted to studies using validated analytical methods [25]. The declining trend in protein concentration of mature milk across the first 3 mo postpartum is consistent with a review of recent studies in which protein content was measured in the mature milk (>21 d) of United States and Canadian mothers [24], as well as with data in the literature [21,[26], [27], [28]].

## Carbohydrate

### Background

Lactose is the principal carbohydrate in human milk, present in concentrations ranging from 67 to 78 g/L [26], followed by human milk oligosaccharides at concentrations of 5–15 g/L [29]. In the infant digestive tract, lactose is broken down into its components, glucose and galactose; in addition to providing energy, galactose is critical for the synthesis of galactolipids that are essential for brain development and myelination [29]. Mammary gland lactose synthesis is a major determinant of the milk volume output, with the concentration of lactose in milk positively associated with milk volume [30]. Lactose concentrations are independent of maternal diet, nutritional status, or BMI [21,31], but increase from colostrum to mature milk to support the energy demands of the infant [3,32].

Although they do not contribute nutritional value, human milk oligosaccharides are prebiotic substrates, encouraging the growth of beneficial intestinal bacteria in the developing infant gut microbiome and serving as decoy receptors for pathogens, among other bioactive functions [3,33]. Nearly 200 unique human milk oligosaccharides with structures varying from 2 to 22 sugars have been identified in human milk [34]. Human milk oligosaccharide concentrations are highest in colostrum and decrease gradually throughout lactation [35]. Total and digestible carbohydrate concentrations are positively associated with infant weight, possibly due to their role in osmolarity and volume regulation [36,37].

### Results

**FIGURE 2:**
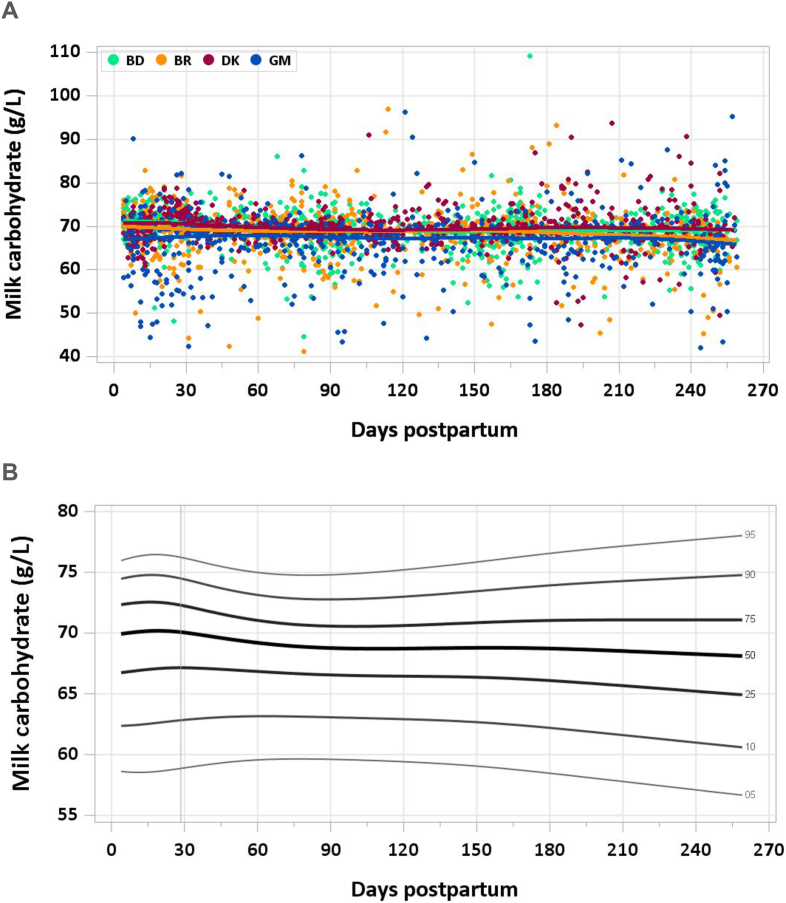
(A) Distribution of human milk carbohydrate concentrations. BD, Bangladesh; BR, Brazil; DK, Denmark; GM, The Gambia. (B) Percentile curves for carbohydrate concentration in human milk.

The median carbohydrate concentration was stable at 68.2–70.1 g/L across the timepoints measured (Figure 2A and B). The pooled median total carbohydrate intake from 1 to 6 mo was 57.1 g/d.

### Comparison with published values

The AI for carbohydrate set by the IOM for infants 0 to 6 mo is 60 g/d, based on an average lactose concentration of 74 g/L (results from 10 studies published between 1981 and 1993) and an estimated milk intake of 0.78 L/d [18]. Although the median carbohydrate concentration of samples in the MILQ and E-MILQ studies was below the concentration used by the IOM, it is consistent with several studies that were cited in the IOM report [[38], [39], [40]].

Notably, the RVs presented in Figure 2A represent total carbohydrates, including ∼20% nonnutritive human milk oligosaccharides [2]. Although the total carbohydrate concentrations likely overestimate the digestible carbohydrate concentrations required for optimal infant growth, total carbohydrate is a more clinically and analytically accessible metric.

## Fat

### Background

Fat is a major source of energy in human milk, as well as the source of essential fatty acids, fat-soluble vitamins, and bioactive species with critical roles in infancy [41]. The complex human milk lipidome includes multiple lipid classes and hundreds of individual lipid species [42]; however, 98%–99% of fat is present in the form of triacylglycerols [41]. Saturated palmitic acid (C16:0) accounts for ∼25% of milk fatty acids and the majority of saturated fatty acid content [43]. The essential fatty acids linoleic acid (LA, C18:2) and α-linolenic acid (ALA, C18:3) are the predominant PUFAs in human milk; both are derived exclusively from the maternal diet either directly or after maternal storage and metabolism [43]. The longer-chain PUFAs arachidonic acid [ARA, C20:4(*n*−6)], EPA [20:5(*n*−3)], and DHA [22:6(*n*−3)] may originate from maternal dietary sources (poultry and other meats for ARA, and fish for EPA and DHA) or be synthesized from LA and ALA in maternal tissues [44]. The biological effects of essential fatty acids in early life, including the development of the brain, eyes, and immune system, are mediated by these longer-chain PUFAs [45]. Both observational and intervention studies have demonstrated considerable variability in concentrations of specific fatty acids in human milk, largely explained by maternal dietary patterns [44]. Interindividual differences in milk fat concentration may also be related to maternal weight or BMI, maternal nutritional status, and genetic, sociodemographic, and environmental factors [11,46].

In addition to interindividual differences in the fatty acid composition of human milk, intraindividual fluctuation is attributed to the fullness of the breast at the point of sampling [47]. Lipid concentration increases as the breast is emptied, ranging from 2% in foremilk to 6% in hindmilk during the first 6 mo of lactation [48]. Though the mechanism regulating fat expression in milk has not been fully elucidated, the higher fat content in hindmilk is due to a higher number of fat globules rather than to larger globules [49]. The MILQ protocols required collecting milk samples by full breast expression to obtain a representative measure of lipid content for the feed.

### Results

**FIGURE 3:**
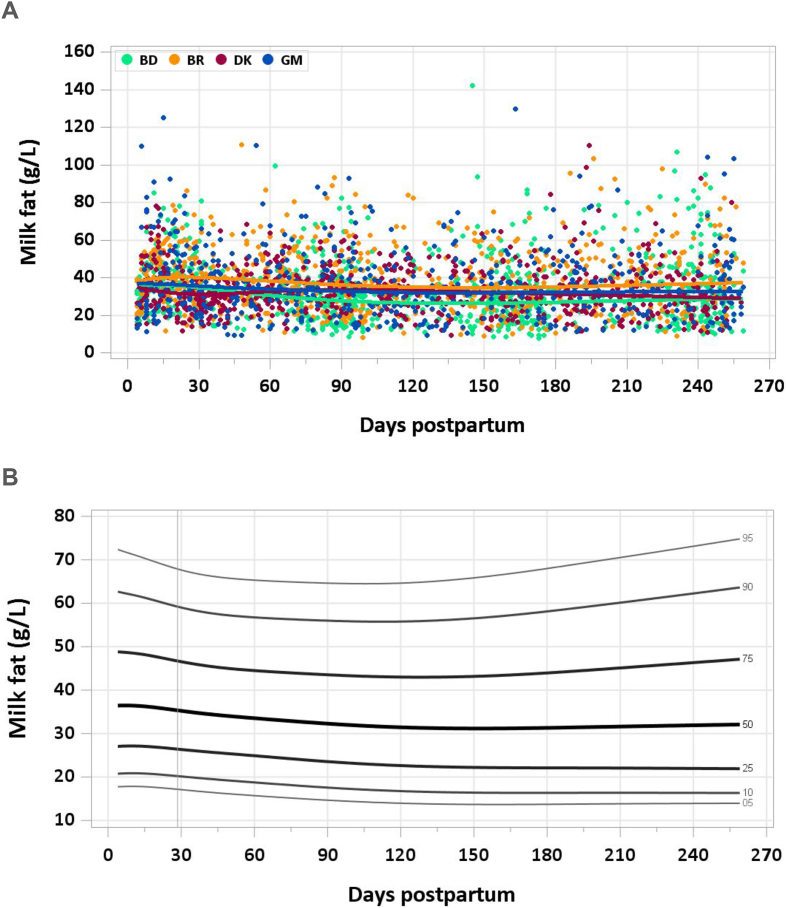
(A) Distribution of human milk fat concentrations. BD, Bangladesh; BR, Brazil; DK, Denmark; GM, The Gambia. (B) Percentile curves for fat concentration in human milk.

The median fat concentration decreased from 37.0 g/L at 4–17 d to 35.9 g/L at 18–31 d and 34.2 g/L at 1–2 mo. By 2–3 mo, median milk fat concentration stabilized in the range of 31.2–32.8 g/L throughout the study period (Figure 3A and B). The pooled median total fat intake from 1 to 6 mo was 26.0 g/d.

### Comparison with published values

The AI for fat set by the IOM for infants 0–6 mo is 31 g/d, based on an average milk fat content of 40 g/L and an estimated intake of 0.78 L/d [18]. The median human milk fat concentration in the present study was lower than the IOM value (32 g/L) and a recent review by Leghi et al. [50] of studies that collected milk by full breast expression. In that review, mean milk fat concentrations were 35.2 ± 3.1 g/L in 8 studies (424 participants) in which milk was collected by full breast expression over a 24 h period and 42.4 ± 13.1 g/L in 6 studies (449 participants) when samples were collected by full breast expression in the morning only. However, in a review that included 21 studies of human milk fat content published between 1992 and 2021, and using various collection protocols, timepoints, and analytical methods, Brockway et al. [4] found a range of 26.0–45.8 g/L total fat, excluding infants with poor weight gain. Neither of these reviews considered maternal diet, BMI, or nutritional status; in the E-MILQ and MILQ studies, an inclusion criterion was maternal prepregnancy or <2 wk postpartum BMI in the range of 18.5–29.9 kg/m^2^ [6].

## Energy

### Background

The energy content of human milk is critical for meeting metabolic needs and supporting infant growth and development during the first months of life. The estimated energy requirements for infants are calculated as the sum of total energy expenditure and the age- and sex-specific energy cost of growth [51]. Total energy expenditures are calculated using predictive equations based on age, weight, and length. For boys, the energy cost of growth is estimated to be 200 kcal/d from 0 to 3 mo and 50 kcal/d for 3–6 mo, averaging 125 kcal/d from 0 to 6 mo. The respective estimates for girls are 180 and 60 kcal/d, with an average of 120 kcal/d [51]. As exclusive breastfeeding is recommended for the first 6 mo of life, it is assumed that energy needs are met through the nutrient-dense and easily digestible components of human milk.

Because of the relative stability of carbohydrates and protein concentration at a given stage of lactation, the energy content of human milk depends largely on variations in fat concentration [52]. Milk volume is inversely related to fat concentration and, therefore, to caloric density [53]. Milk composition adapts dynamically according to need; preterm milk has higher energy density (700–800 kcal/L) to support the catch-up growth of infants born prematurely [54].

### Methods

The energy concentration of milk was calculated using a near-infrared spectroscopy instrument based on the concentrations of protein, carbohydrate, and fat.

### Results

**FIGURE 4:**
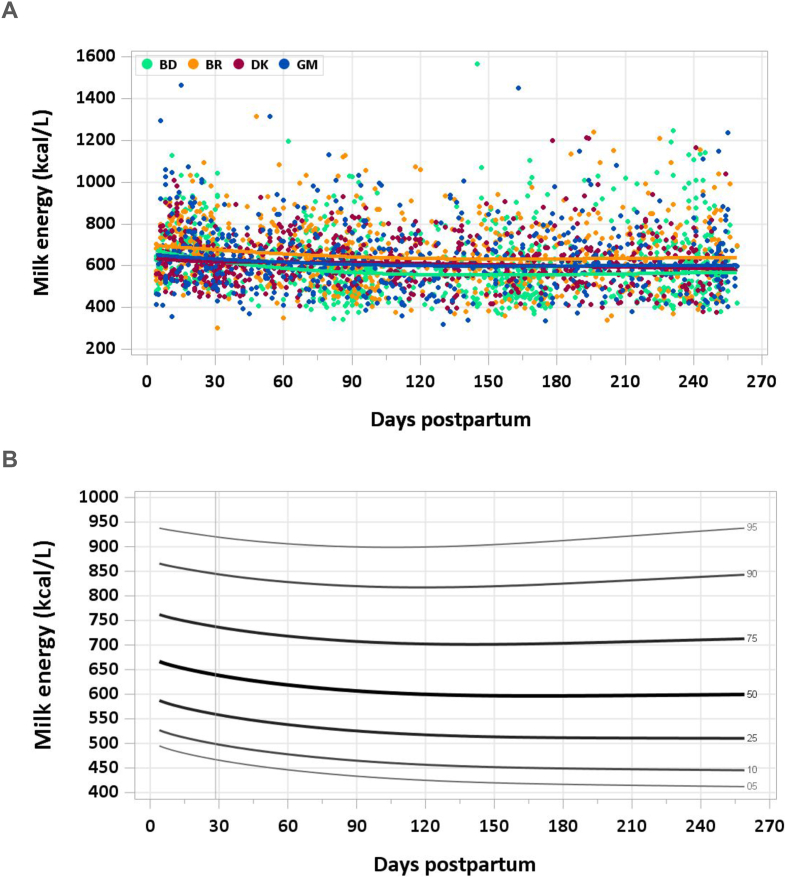
(A) Distribution of human milk energy content. BD, Bangladesh; BR, Brazil; DK, Denmark; GM, The Gambia. (B) Percentile curves for energy content in human milk.

Energy density was highest in early milk, with a median of 665 kcal/L at 4–17 d and 650 kcal/L at 18–31 d. From 3 to 4 mo until the end of the study period, the 50th percentile of energy density was stable at 597–602 kcal/L. The early decrease in protein and fat concentration was echoed in the energy curve (Figure 4A and B). The pooled median energy intake from 1 to 6 mo was 487.5 kcal/d.

### Comparison with published values

The NAM assumes an average energy density of human milk of 650 kcal/L and estimates that an exclusively breastfed infant consuming 0.78 L/d consumes ∼500 kcal/d [51]. The estimated energy requirement from 0 to 6 mo is based on age and weight, and using reference average weights for each sex, is estimated in the range of 438–645 kcal/d. The American Academy of Pediatrics references the energy density of human milk as 650–700 kcal/L [55]. The energy density of human milk measured in the present study was lower than the NAM and American Academy of Pediatrics values and a published study reporting a mean of 697 ± 67 kcal/L in the United States [21], but consistent with another study reporting a geometric mean of 568 kcal/L (95% CI: 542, 619) in Cambodia [37].

## Discussion

The primary objective of the MILQ study was to develop RVs for nutrient concentrations in the milk of well-nourished mothers during the first 8.5 mo of lactation. Results suggest that milk macronutrient concentrations used by the IOM to develop infant intake recommendations were 35% and 25% higher for protein and fat, respectively, than median concentrations measured in the MILQ study. This discrepancy was more subtle for carbohydrate and energy, where milk nutrient concentrations used to develop intake recommendations were 7.5% above the MILQ median concentration. It is notable that concentrations used by the IOM represent the period from 0 to 6 mo whereas MILQ estimates are from 1 to 6 mo, excluding the earliest period of lactation when protein and fat concentrations are highest.

In the MILQ study, milk volume quantified by deuterium oxide dose to mother (or test weighing in Denmark) together with milk nutrient concentration enabled the calculation of total nutrient intake by the infant at multiple time points over the first 8.5 mo of lactation. The pooled median infant daily intakes of protein, carbohydrate, and fat from 1 to 6 mo in the MILQ study were 65%, 95% and 84% of the AIs, respectively. There is no AI for energy, as the requirement depends on sex and weight.

Strengths and limitations of the MILQ and E-MILQ studies are discussed in the introduction and study design article [6] in this supplement. The study design was unique in systematically collecting milk from well-nourished women in diverse geographical regions over time points representative of the comprehensive period through 8.5 mo of lactation, and measuring milk volume transferred to the infant in tandem with nutrient concentration.

In conclusion the MILQ and E-MILQ studies provide RVs for protein, carbohydrate, fat, and energy concentrations in human milk across the first 8.5 mo of lactation from a dataset of well-nourished women from 4 country sites. Although milk macronutrient composition varies among women due to biological and environmental factors, the focus of the E-MILQ and MILQ data presented here is to demonstrate the distribution of nutrient concentrations and trends across time and study sites. The data confirm a decline in protein and fat concentrations during early lactation, stabilizing in later months, whereas carbohydrate concentrations remain relatively consistent throughout the study period. Energy density mirrors the trends observed for fat, reflecting its central role in caloric variability.

Median human milk concentrations of carbohydrates and energy in the MILQ study were consistent with existing values, and median total daily intakes were on par with the published AIs. For protein and fat, median human milk nutrient concentrations and total daily intakes from MILQ were lower than those suggested by the IOM (∼65% of IOM values for protein and ∼80% of IOM values for fat), though it is notable that IOM recommendations include the period from 0 to 1 mo, when fat and protein concentrations are highest. The presented RVs offer a valuable resource for understanding human milk composition in diverse populations and can inform public health recommendations to support optimal infant growth and development.

## Author contributions

The authors’ responsibilities were as follows – LHA, GK, MMI, SEM, DH, CM: designed research; DH, DdBM, ACF, ADdSC, MH, SHC, JIL: conducted research; DH, JMP: analyzed data; JIL, DKD: wrote the paper; LHA: had primary responsibility for the final content; and all authors: read and approved the final manuscript.

## Data availability

Data described in the manuscript, code book, and analytic code will be made available upon request pending application and approval.

## Funding

The publication of this supplement is supported by the Gates Foundation (OPP1148405/INV-002300, OPP1061055) and USDA intramural funds (2023-51530-025-00D).

## Conflict of interest

The authors report no conflicts of interest.
