# Supplementary material for: Reference Values for Macronutrients in Human Milk: the Mothers, Infants and Lactation Quality (MILQ) Study
Source: Adv Nutr. 2025 Oct 28;16(Suppl 1):100501. doi: 10.1016/j.advnut.2025.100501 (PMC12673390; doi:10.1016/j.advnut.2025.100501)
Supplement: Multimedia component 1 [file mmc1.docx]

**Supplementary Table 1.** Monthly percentile summaries for macronutrient concentration in human milk

| Protein (g/L) | **Age** | **P05** | **P10** | **P25** | **P50** | **P75** | **P90** | **P95** |
| --- | --- | --- | --- | --- | --- | --- | --- | --- |
|  |  |  |  |  |  |  |  |  |
|  | 4-17 d | 8.25 | 9.36 | 10.89 | 12.46 | 14.27 | 16.46 | 18.21 |
|  | 18-31 d | 6.93 | 7.92 | 9.29 | 10.71 | 12.39 | 14.50 | 16.21 |
|  | 1-2 m | 6.27 | 7.20 | 8.49 | 9.81 | 11.39 | 13.39 | 15.04 |
|  | 2-3 m | 5.60 | 6.44 | 7.61 | 8.85 | 10.40 | 12.47 | 14.21 |
|  | 3-4 m | 5.20 | 5.97 | 7.03 | 8.21 | 9.77 | 11.94 | 13.83 |
|  | 4-5 m | 5.01 | 5.73 | 6.74 | 7.89 | 9.46 | 11.72 | 13.72 |
|  | 5-6 m | 4.89 | 5.62 | 6.62 | 7.76 | 9.36 | 11.69 | 13.78 |
|  | 6-7 m | 4.83 | 5.57 | 6.59 | 7.75 | 9.37 | 11.75 | 13.90 |
|  | 7-8 m | 4.80 | 5.56 | 6.60 | 7.77 | 9.42 | 11.84 | 14.05 |
|  | 8-8.5 m | 4.78 | 5.56 | 6.62 | 7.80 | 9.46 | 11.92 | 14.15 |
|  |  |  |  |  |  |  |  |  |
|  |  |  |  |  |  |  |  |  |
| Carbohydrate (g/L) | **Age** | **P05** | **P10** | **P25** | **P50** | **P75** | **P90** | **P95** |
|  |  |  |  |  |  |  |  |  |
|  | 4-17 d | 58.6 | 62.5 | 66.9 | 70.1 | 72.5 | 74.7 | 76.3 |
|  | 18-31 d | 58.8 | 62.7 | 67.1 | 70.1 | 72.4 | 74.6 | 76.3 |
|  | 1-2 m | 59.3 | 63.1 | 67.0 | 69.6 | 71.6 | 73.7 | 75.5 |
|  | 2-3 m | 59.6 | 63.1 | 66.7 | 68.9 | 70.7 | 72.9 | 74.8 |
|  | 3-4 m | 59.5 | 63.0 | 66.5 | 68.7 | 70.6 | 72.9 | 75.0 |
|  | 4-5 m | 59.2 | 62.8 | 66.4 | 68.8 | 70.8 | 73.2 | 75.6 |
|  | 5-6 m | 58.7 | 62.4 | 66.2 | 68.8 | 71.0 | 73.7 | 76.3 |
|  | 6-7 m | 58.1 | 61.9 | 65.9 | 68.6 | 71.1 | 74.2 | 76.9 |
|  | 7-8 m | 57.4 | 61.2 | 65.4 | 68.4 | 71.1 | 74.5 | 77.5 |
|  | 8-8,5 m | 56.8 | 60.8 | 65.0 | 68.2 | 71.1 | 74.7 | 77.9 |
|  |  |  |  |  |  |  |  |  |
|  |  |  |  |  |  |  |  |  |
| Fat (g/L) | **Age** | **P05** | **P10** | **P25** | **P50** | **P75** | **P90** | **P95** |
|  |  |  |  |  |  |  |  |  |
|  | 4-17 d | 17.8 | 20.8 | 27.1 | 36.4 | 48.5 | 61.9 | 71.2 |
|  | 18-31 d | 17.4 | 20.4 | 26.6 | 35.6 | 47.2 | 59.8 | 68.6 |
|  | 1-2 m | 16.3 | 19.4 | 25.6 | 34.3 | 45.3 | 57.5 | 66.1 |
|  | 2-3 m | 15.1 | 18.1 | 24.2 | 32.9 | 44.0 | 56.3 | 64.9 |
|  | 3-4 m | 14.3 | 17.1 | 23.0 | 31.8 | 43.2 | 55.8 | 64.5 |
|  | 4-5 m | 13.8 | 16.5 | 22.4 | 31.3 | 43.0 | 56.1 | 65.2 |
|  | 5-6 m | 13.7 | 16.4 | 22.1 | 31.2 | 43.6 | 57.4 | 67.0 |
|  | 6-7 m | 13.8 | 16.4 | 22.1 | 31.5 | 44.6 | 59.3 | 69.5 |
|  | 7-8 m | 13.9 | 16.4 | 22.0 | 31.8 | 45.9 | 61.4 | 72.1 |
|  | 8-8.5 m | 14.0 | 16.3 | 21.9 | 32.0 | 46.8 | 63.1 | 74.1 |
|  |  |  |  |  |  |  |  |  |
|  |  |  |  |  |  |  |  |  |
| Energy density (kcal/L) | **Age** | **P05** | **P10** | **P25** | **P50** | **P75** | **P90** | **P95** |
|  |  |  |  |  |  |  |  |  |
|  | 4-17 d | 486 | 518 | 578 | 658 | 754 | 859 | 932 |
|  | 18-31 d | 471 | 503 | 563 | 643 | 741 | 848 | 923 |
|  | 1-2 m | 455 | 486 | 547 | 627 | 726 | 835 | 911 |
|  | 2-3 m | 439 | 471 | 531 | 612 | 712 | 823 | 902 |
|  | 3-4 m | 428 | 460 | 521 | 603 | 704 | 818 | 899 |
|  | 4-5 m | 422 | 454 | 515 | 598 | 702 | 818 | 902 |
|  | 5-6 m | 418 | 450 | 512 | 597 | 703 | 823 | 909 |
|  | 6-7 m | 416 | 448 | 511 | 597 | 706 | 829 | 918 |
|  | 7-8 m | 414 | 447 | 511 | 598 | 709 | 836 | 928 |
|  | 8-8.5 m | 413 | 446 | 510 | 599 | 712 | 841 | 935 |

**Supplementary Table 2**. Median infant macronutrient intake by study visit

|  | **1-3.49 mo** | **3.5-5.99 mo** | **6-8.5 mo** |
| --- | --- | --- | --- |
| Protein (g/d) | 7.424 | 6.462 | 5.38 |
| Carbohydrate (g/d) | 54.62 | 56.94 | 48.28 |
| Fat (g/d) | 26.03 | 25.51 | 21.72 |
| Energy (kcal/d) | 488.4 | 483.3 | 418.7 |
